# Effect of intravenous lidocaine on the ED50 of propofol induction dose in elderly patients undergoing painless gastroscopy: a prospective, randomized, double-blinded, controlled study

**DOI:** 10.1186/s13741-024-00370-2

**Published:** 2024-03-05

**Authors:** Lili Tang, Wenhui Lv, Jingjing Sun, Lijian Chen

**Affiliations:** Department of Anesthesiology, the First Affiliated Hospital of Anhui Medical University, Hefei, Anhui China

**Keywords:** Lidocaine, Propofol, Median effective dose, Elderly patients, Painless gastroscopy

## Abstract

**Background:**

Intravenous lidocaine could be a potential alternative adjuvant to propofol-based sedation for gastroscopy in elderly patients. This study aimed to evaluate the effect of intravenous lidocaine on the median effective dose (ED50) of propofol induction dose in elderly patients undergoing painless gastroscopy.

**Methods:**

The study included 70 patients aged ≥ 60 years undergoing painless gastroscopy with 64 randomly assigned to either group L (2% lidocaine 1.5 mg/kg, *n* = 31) or group N (equal volume normal saline, *n* = 33). All patients received propofol induction following 0.1 μg/kg intravenous sufentanil. The Dixon “up-and-down” sequential method was used, with a 1.5 mg/kg initial induction dose of propofol followed by a 0.1 mg/kg sequential variable dose. The primary endpoint was the ED50 of the propofol induction dose. The total propofol dose, recovery time, adverse events, and local anesthetic intoxication reactions were also recorded.

**Results:**

The ED50 of propofol induction dose was 0.670 (95% confidence interval [CI] 0.216–0.827) mg/kg in group L and 1.118 (95% CI 0.803–1.232) mg/kg in group N. There was a statistically significant difference between the two groups (*p* < 0.001). The incidence of hypotension and propofol injection pain were lower in group L than in group N (*p* < 0.05). Furthermore, the orientation recovery time in group L was shorter compared to group N (*p* < 0.05). None of the participants in group L observed local anesthetic intoxication reactions after receiving lidocaine.

**Conclusions:**

The administration of intravenous lidocaine to elderly patients undergoing painless gastroscopy resulted in a significant 40% reduction in the ED50 of propofol induction dose, which may be related to the decreased incidence of hypotension and injection pain, as well as the improved post-gastroscopy orientation recovery.

**Trial registration:**

ChiCTR, ChiCTR2200065530. Registered on 08 November 2022.

## Introduction

Gastroscopy screening is an essential strategy to improve the overall 5-year survival rates by more than 50% for esophageal and gastric cancers (Katai et al. 2017; Xia et al. 2021), which account for approximately 50% of the global burden in China (Sung et al. 2021). Propofol, an ultrashort-acting intravenous anesthetic with rapid onset and fast recovery, in combination with an opioid analgesic, is the most commonly used anesthesia strategy for a painless gastroscopy (Luginbühl et al. 2009; Zhou et al. 2021). Nevertheless, propofol can adversely affect cardiovascular and respiratory functions in a dose-dependent manner (Coté et al. 2010; Landoni et al. 2013), especially in elderly patients (Khoi et al. 2015).

Considering the increasing incidence of upper gastrointestinal tract cancer in the aging population (Liang et al. 2013), gastroscopy screening can be quite beneficial. However, due to age-related physiological changes, such as deteriorating organ function, variability in pharmacokinetics and pharmacodynamics, and the presence of comorbidities, anesthesia management for elderly patients undergoing gastroscopy remains challenging (Geng et al. 2018). Evidence suggests that advancing age and higher loading doses of propofol are associated with increased rates of sedation-related adverse events (Khoi et al. 2015). Therefore, it is crucial to incorporate an adjuvant agent with propofol to minimize the required dosage and mitigate adverse events to ensure the safety of elderly patients.

Lidocaine, a commonly used local anesthetic and anti-arrhythmia agent, has displayed promising results as an adjuvant to propofol-based sedation when administered intravenously. Previous researches have demonstrated that intravenous lidocaine effectively reduces propofol consumption, alleviates visceral pain, lowers the occurrence of hypoxia, and promotes faster recovery of bowel function following surgical and endoscopic procedures (Forster et al. 2018; Gross et al. 1983; Kaba et al. 2007; Song et al. 2017). However, there is little information on the use of intravenous lidocaine in combination with propofol in elderly patients (Hu et al. 2022). Consequently, the minimum effective dose of propofol when coupled with lidocaine for this specific population undergoing gastroscopy has not yet been defined. Therefore, the purpose of this study was to determine the median effective dose (ED50) of propofol induction dose when combined with intravenous lidocaine, as well as to investigate any potential adverse events such as cardiopulmonary complications, injection pain, and postoperative recovery in elderly patients undergoing painless gastroscopy.

## Methods

### Study setting

This was a prospective, randomized, controlled, double-blind trial that was approved by the Ethical Committee of the First Affiliated Hospital of Anhui Medical University, Hefei, China (Approval No. PJ2022-09–43) and was registered in the Chinese Clinical Trial Registry (ChiCTR2200065530, date: 08 November 2022). This study was conducted in the Endoscopic Unit of the First Affiliated Hospital of Anhui Medical University, spanning from January 2023 to June 2023. Written informed consents were obtained from all participating patients.

### Patients

We recruited elderly patients scheduled for painless gastroscopy. All patients were aged ≥ 60 years, American Society of Anesthesiologists (ASA) physical status II or III, and a body mass index (BMI) between 18 and 24 kg/m^2^. The exclusion criteria were as follows: severe cardiac arrhythmia; epilepsy; severe dysfunction in the liver and kidney; history of alcohol abuse or drug dependence; history of allergy to soy, milk, propofol, sufentanil, or local anesthetic drugs; and refusal to provide an informed consent form.

### Randomization and blinding

Patients were randomly divided into the lidocaine group (group L) and the normal saline group (group N), using a random number table at a 1:1 ratio. The allocation details were concealed using opaque sealed envelopes. A 2% lidocaine at a dosage of 1.5 mg/kg or an equal volume of 0.9% normal saline was prepared in a 20-ml syringe according to the assigned patient groups by a nurse who was not involved in the investigation. The syringes containing the solutions were unlabeled and handed over to an anesthesiologist who administered the medicines, performed general anesthesia, and recorded perioperative data. The anesthesiologist, patients, and endoscopists, were unaware of the group assignment and the contents of the syringes.

### Study protocol

All patients were routinely fasted for at least 8 h for solids and 2 h for water. Upon entering the examination room, the patient was positioned on the examination bed in the left lateral position, and peripheral vascular access was established in the right upper limb. Electrocardiography, noninvasive blood pressure, and pulse oximetry were continuously monitored. Patients were administered 100% oxygen using a mask at a 4–6 L/min flow rate.

A single bolus of 2% lidocaine at a dosage of 1.5 mg/kg or an equal volume of normal saline was administered by gradual intravenous injection. After a 2-min interval, anesthesia induction was initiated by administering propofol within 60 s after administering 0.1 μg/kg intravenous sufentanil. The level of sedation was assessed using the Modified Observer’s Assessment of Alertness/Sedation Scale (MOAA/S) (Cohen et al. 2007), which ranges from 5 (responds readily to name spoken in normal tone) to 0 (no response after painful trapezius squeeze). Gastroscopy was performed by the endoscopist when the patient’s MOAA/S score was ≤ 1. The initial induction dose of propofol was set at 1.5 mg/kg for both groups. The Dixon “up-and-down” sequential method (Dixon 1991; Pace et al. 2007) was utilized to determine the dosage of the subsequent patient, with a sequential variable dose of 0.1 mg/kg. That is, if the first enrolled patient coughed or moved during gastroscope implantation after anesthesia induction (defined as ineffective sedation), the propofol dosage for the next patient would be increased by a dose grade of 0.1 mg/kg. Conversely, if the first patient did not cough or move during gastroscope implantation after anesthesia induction (defined as effective sedation), the induction dose for the next patient would be decreased by one dose grade. The Dixon method required at least six pairs of ineffective/effective sedation episodes to determine the ED50 of propofol induction dose. In this study, seven crossover sites were considered sufficient for this purpose. If a patient coughed or moved during the gastroscopy procedure, an additional dose of 0.5–1.0 mg/kg of propofol was administered as a rescue medication.

Intraoperative monitoring was performed to maintain the patient’s heart rate (HR) between 45 and 100 beats/min, mean arterial pressure (MAP) fluctuations within 20% of the baseline value, and pulse oxygen saturation (SpO_2_) levels between 92 and 100%. If the HR dropped below 45 beats/min, 0.3–0.5 mg of atropine was administered intravenously. Hypotension, defined as a decrease in MAP exceeding 20% of the baseline value, was treated immediately with 3–6 mg dose of ephedrine. If respiratory depression was detected, defined as a minimum SpO_2_ level below 92%, measures were taken to increase oxygen flow, adjust the mandible position, and provide face mask ventilation if necessary.

### Outcome* measurements*

The primary endpoint was the ED50 of the propofol induction dose. Secondary endpoints included the added and total doses of propofol, the procedure time (from insertion to withdrawal of the endoscopic), the awakening time (from endoscopic withdrawal to opening eyes), the orientation recovery time (from endoscopic withdrawal to answering questions about name and location), duration of stay in postanesthesia care unit (PACU, from endoscopic withdrawal to reaching a Steward score of 6), common adverse events such as hypotension, respiratory depression, propofol injection pain, nausea-vomiting, and local anesthetic intoxication reactions of cardiotoxic (e.g., increased intervals or widened QRS complex) or neurotoxic (e.g., dizziness, drowsiness, oral metal odor, mouth paresthesia, blurred vision, or convulsion). Additionally, HR, MAP, SpO_2_, and respiratory rate (RR) were also measured at baseline (T0), after induction (T1), at the end of gastroscopy (T2), and during awakening (T3).

### Statistical analysis

Statistical analyses were performed using IBM SPSS Statistics version 25.0. Normality of the quantitative data was tested, and normally distributed variables were presented as mean ± standard deviation (SD) and compared using independent-sample *t*-tests. Non-normally distributed continuous variables were reported as medians with interquartile ranges (IQR) and analyzed using nonparametric Mann–Whitney* U* tests. Categorical variables were expressed as numbers and percentages (%) and tested using chi-square tests or Fisher’s exact tests. Changes in hemodynamic and respiratory variables were analyzed using the repeated measures analysis of variance (ANOVA). The ED50 and 95% confidence interval (CI) of propofol were calculated using the probit method (probability unit regression). GraphPad Prism version 8.0 was used for data visualization. Statistical significance was defined as a *P* value < 0.05.

## Results

**Fig. 1:**
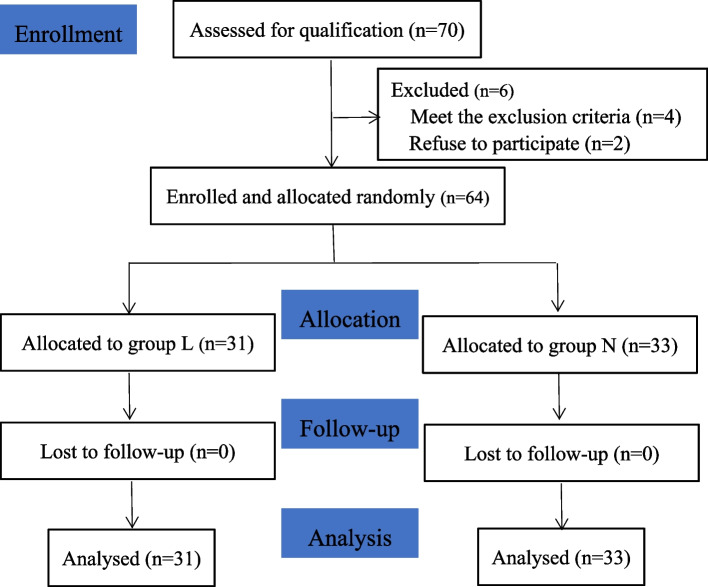
Consolidated standards of reporting trials flow chart

A total of 70 patients were initially assessed for eligibility. However, six patients were excluded: four due to meeting the exclusion criteria and two refusing to participate. Finally, 64 patients were enrolled and randomly allocated into two groups. The groups consisted of 31 patients in group L and 33 patients in group N, achieving the required six ineffective/effective pairs for analysis (Fig. 1).

**Fig. 2:**
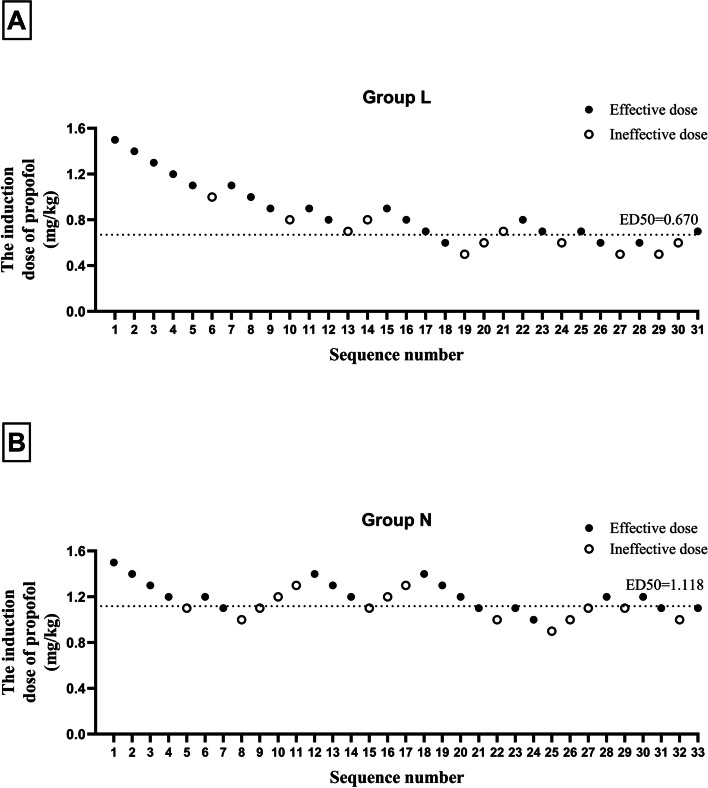
Sequential induction dose adjustment of propofol by the Dixon method in two groups of elderly patients undergoing painless gastroscopy. **A** Sequential induction dose of propofol when combined with 2% lidocaine 1.5 mg/kg in group L of elderly patients undergoing painless gastroscopy. The ED50 value was 0.670 mg/kg in group L. **B** Sequential induction dose adjustment of propofol in group N of elderly patients undergoing painless gastroscopy. The ED50 value in group N was 1.118 mg/kg. *ED50* median effective dose

**Fig. 3:**
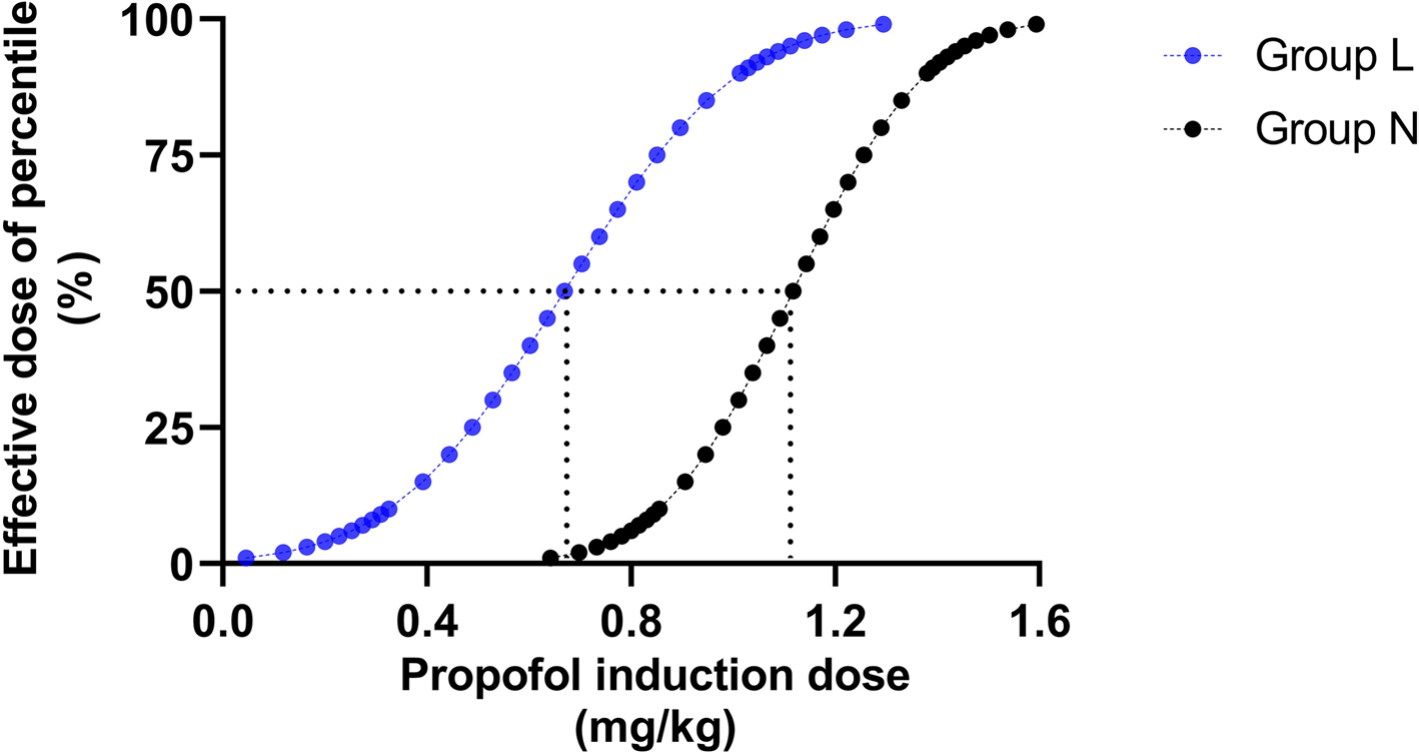
The dose–response curve of propofol induction dose in two groups

Demographic characteristics, including age, sex, height, body weight, and comorbidity, were not significantly different between the two groups. The groups had comparable procedure times, awakening times, and duration of stay in PACU; the inspection types were identical (*p* > 0.05). However, the orientation recovery time in group L was shorter than that in group N (1.00 [1.00, 1.83] min vs. 1.50 [1.00, 3.00] min, *p* = 0.047) (Table 1). The ED50 of propofol induction dose, determined by the Dixon up-and-down sequential method, was 0.670 (95% CI 0.216–0.827) mg/kg in group L and 1.118 (95% CI 0.803–1.232) mg/kg in group N. The ED50 value significantly differed between the two groups (*p* < 0.001) (Table 2). The Dixon up-and-down sequences and the dose–response analysis of lidocaine coadministered with propofol on patient responses demonstrated that the propofol induction dose in group L was significantly lower than that in group N (Figs. 2 and 3). The sufentanil consumption did not differ significantly between the two groups (*p* > 0.05).

**Table 1:** The demographic characteristics and perioperative profiles between the two groups

Demographic data	Group L (*n* = 31)	Group N (*n* = 33)	*P* value
Age (year)	69.13 ± 6.13	68.12 ± 5.44	0.489
Sex, *n* (%)			0.981
Male	14(45.16%)	15(45.45%)	
Female	17(54.83%)	18(54.54%)	
Height (cm)	162.87 ± 6.95	161.58 ± 7.65	0.482
Weight (kg)	58.23 ± 6.92	56.52 ± 8.18	0.371
BMI (kg/m^2^)	21.91 ± 1.75	21.54 ± 1.72	0.404
ASA class, *n* (%)			0.468
II	26(83.87%)	30(90.91%)	
III	5(16.13%)	3(9.68%)	
Comorbidities, *n* (%)			
Hypertension	10(32.26%)	7(21.21%)	0.317
Diabetes	4(12.9%)	2(6.06%)	0.419
Cardiovascular disease	4(12.9%)	1	0.190
Cerebrovascular disease	1	0	0.484
Inspection time (min)	3.92(3.00, 4.58)	4.33(3.50, 5.67)	0.089
Time of awakening (s)	10(10, 30)	10(3.5, 60)	0.815
Time of orientation recovery (min)	1.00(1.00, 1.83)	1.50(1.00, 3.00)	0.047*
Time of staying in PACU (min)	20(19, 22)	20(18, 21)	0.247
Type of inspection, *n* (%)			0.374
Examination	21(67.74%)	25(75.76%)	
Polypectomy	1(3.23%)	3(9.09%)	
Biopsy	9(29.03%)	5(15.15%)	

*BMI* body mass index, *ASA* American Society of Anesthesiologists, *PACU* postanesthesia care unit

^*^p < 0.05

**Table 2:** The consumption of propofol, sufentanil, and lidocaine

	Group L (*n* = 31)	Group N (*n* = 33)	*P* value
Propofol			
Induction dose (mg)	43.2(34.8, 57.0)	63.8(57.4, 77.4)	< 0.001 *
Added dose (mg)	0(0, 20)	0(0, 20)	0.539
Total dose (mg)	56.45 ± 17.38	77.32 ± 14.25	< 0.001 *
The ED50 of propofol induction dose (mg/kg) (95%CI)	0.670 (0.216–0.827)	1.118 (0.803–1.232)	< 0.001 *
Sufentanil (μg)	5.83 ± 0.69	5.65 ± 0.82	0.363
Lidocaine (mg)	87.44 ± 10.39	–	–

*ED50* Median effective dose, *CI* Confidence interval

^*^p < 0.05

Totally 6 patients in group L and 14 patients in group N experienced hypotension, with a difference between the two groups (19.35% vs. 42.42%, *p* = 0.047). The incidence of propofol injection pain was 63.64% in group N, which was higher than the 29.03% observed in group L (*p* = 0.008). However, there were no significant differences in the incidence of respiratory depression and nausea-vomiting between the two groups (*p* > 0.05). None of the participants in group L exhibited any reactions related to local anesthetic intoxication after receiving lidocaine (Table 3).

**Table 3:** The common side effects and local anesthetic intoxication reactions between the two groups

	Group L (*n* = 31)	Group N (*n* = 33)	*P* value
Hypotension, *n* (%)	6(19.35%)	14(42.42%)	0.047*
Respiratory depression, *n* (%)	1(3.23%)	5(15.15%)	0.198
Propofol injection pain, *n* (%)	9(29.03%)	21(63.64%)	0.008*
Nausea-vomiting, *n* (%)	0	2(6.06%)	0.493
Local anesthetic intoxication reactions, *n* (%)	0	–	–

Values are expressed as the number of patients and percent

^*^p < 0.05

In terms of hemodynamic and respiratory parameters, group L had higher values of MAP at T1 and SpO_2_ at T2 than group N (*p* < 0.05). There were no differences between the two groups at other time points. HR and RR were not significantly different between the groups. Within the group, compared with T0, MAP, HR, and RR were lower at various time points, whereas SpO_2_ showed no significant differences at different time points (Table 4).

**Table 4:** Comparison of hemodynamic and respiratory parameters between the two groups at different time points

		Group L (*n* = 31)	Group N (*n* = 33)	*P* value
MAP	T0	89.94 ± 5.20	89.21 ± 5.17	0.579
	T1	80.03 ± 10.93	70.79 ± 8.62	0.000*
	T2	82.52 ± 12.36	79.88 ± 8.62	0.323
	T3	85.58 ± 11.70	84.76 ± 7.79	0.740
HR	T0	80.97 ± 13.42	78.18 ± 14.78	0.434
	T1	76.06 ± 10.94	72.45 ± 11.90	0.212
	T2	73.68 ± 12.82	70.70 ± 11.13	0.324
	T3	75.26 ± 12.33	72.82 ± 10.47	0.396
SpO_2_	T0	99(98, 100)	98(96.5, 100)	0.139
	T1	100(100, 100)	100(99.5, 100)	0.821
	T2	100(99, 100)	99(98, 100)	0.035*
	T3	99(98, 100)	99(98, 99)	0.413
RR	T0	15(15, 16)	16(15, 16)	0.053
	T1	11(11, 12)	12(11, 14)	0.054
	T2	11(11, 11)	11(11, 12)	0.175
	T3	14(12, 15)	14(13, 15)	0.230

Values are expressed as the mean ± standard deviation or medians (IQR). Compared with group N

*MAP* Mean arterial pressure, *HR* Heart rate, *SpO*_*2*_ Pulse oxygen saturation, *RR* Respiratory rate. *T0* the baseline value, *T1* after induction, *T2* the end of gastroscopy, *T3* awakening

^*^p < 0.05

## Discussion

To the best of our knowledge, this study was the first randomized dose–response study to investigate the effect of intravenous lidocaine on the ED50 of propofol induction dose in elderly patients undergoing painless gastroscopy. The results of this study revealed several significant findings. Firstly, the administration of 2% lidocaine at a dose of 1.5 mg/kg resulted in a significant 40% reduction in the ED50 of propofol induction dose (0.670 mg/kg vs. 1.118 mg/kg) in elderly patients undergoing painless gastroscopy. Secondly, the use of intravenous lidocaine was associated with a decreased incidence of hypotension and propofol injection pain, as well as improved post-procedure orientation recovery. Importantly, no lidocaine-related local anesthetic intoxication reactions were observed during the study.

Discomfort associated with gastroscopy results primarily from the stimulation of the oropharynx secondary to the insertion of endoscopic produce. This can lead to mechanical obstruction of the pharynx or compression of the trachea, triggering the cough reflex. Surprisingly, a previous study has demonstrated that intravenous lidocaine at a dose of 1.5 mg/kg or higher effectively suppresses the cough reflex during tracheal intubation (Abernethy et.al. 1983). Accordingly, 1.5 mg/kg of lidocaine intravenously has been shown to reduce the induction dose of propofol by 27% during tracheal intubation in younger adults (Kelsaka et al. 2011). Expanding on these observations, we sought to administer lidocaine during propofol-based sedation to reduce the consumption of propofol secondary to restrain coughing or physical movement during gastroscope implantation in elderly patients. Our study found that the ED50 of propofol induction dose in elderly patients undergoing painless gastroscopy was 0.670 mg/kg when combined with intravenous lidocaine, a significant 40% reduction from the ED50 of 1.118 mg/kg without lidocaine. The finding regarding the propofol-sparing effect of intravenous lidocaine was consistent with previous studies (Chen et al. 2020; Hu et al. 2022; Li et al. 2022). The mechanism underlying this propofol-sparing effect may be attributed to the anti-nociceptive stimulus of lidocaine in this patient group(Hans et al. 2010). Obviously, the ED50 of propofol induction dose in our study was significantly lower than the aforementioned studies, which employed dosages ranging from 1.0 to 1.5 mg/kg. This difference may be due to the Dixon “up-and-down” sequential method used in our study to determine the ED50 of propofol induction dose, which exposes patients to only the minimal effective dose.

The increased vulnerability to hypotension and respiratory depression during propofol-based sedation for painless endoscopy in elderly patients is one of the major concerns. Our goal in administering lidocaine as an adjuvant to propofol was to determine the minimum effective induction dose of propofol and subsequently minimize its adverse effects in this particular population. In our study, the incidence of hypotension was reduced by more than 23% through the use of lidocaine. Both groups showed a decrease in MAP after anesthesia induction (T1) compared with baseline values (T0); however, the lidocaine group experienced a smaller decline. These findings are consistent with previous research by Chen et al., which indicated that combining lidocaine with propofol improved hemodynamic stability in elderly patients during endoscopy procedures (Chen et al. 2020). Despite the significant propofol-sparing effect and the potentially enhanced ventilatory response to CO_2_ produced by lidocaine (Gross et al. 1983), we did not observe a significant difference in the incidence of respiratory depression between group L and group N (3.25% vs. 15.15%). Pain during propofol injection is the most distressing part of the perioperative period, with an overall risk of approximately 60% in untreated patients (Jalota et al. 2011). Our study demonstrated a lower incidence of injection pain in group L compared to group N (29.03% vs. 63.64%). This finding aligned with a meta-analysis suggesting that intravenous lidocaine is the most promising strategy for reducing propofol injection pain (Euasobhon et al. 2016). Additionally, intravenous lidocaine showed a positive effect in promoting orientation recovery after gastroscopy procedures. However, we did not observe other potential benefits of intravenous lidocaine in terms of awakening time, time of stay in PACU, or the incidence of nausea-vomiting.

According to the Enhanced Recovery After Surgery guidelines for gastrointestinal surgery in 2016 (Feldheiser et al. 2015), the recommended dosage of lidocaine was 1.5 mg/kg. Importantly, all elderly patients in our study were monitored closely after lidocaine administration, and no lidocaine-related adverse reactions were observed throughout the perioperative period. Previous studies have shown that the plasma concentrations of lidocaine at this dosage in the elderly population were significantly lower than the levels associated with cardiotoxicity and neurotoxicity, affirming its safety in this population (Abernethy et.al. 1983).

There are several limitations to consider in our study. First, there may be some bias in assessing the depth of sedation as we relied solely on the MOAA/S score, rather than using more objective indicators such as bispectral index or electroencephalogram monitoring. Second, we did not measure the plasma concentration of lidocaine, which could provide more precise information about its pharmacokinetics. Furthermore, the Dixon “up-and-down” sequential method used in our study was neither designed nor powered to statistically evaluate cardiopulmonary adverse effects. Therefore, further research with larger sample sizes and multicenter trials is warranted to draw definitive conclusions on the potential benefits of the propofol-sparing effect of lidocaine.

## Conclusions

In elderly patients undergoing painless gastroscopy, administering 2% lidocaine intravenously at a dosage of 1.5 mg/kg resulted in a significant 40% reduction in the ED50 of propofol induction dose. Furthermore, intravenous lidocaine as an adjuvant to propofol-based sedation led to a decreased incidence of injection pain and hypotension, as well as improved post-gastroscopy orientation recovery without any lidocaine-related local anesthetic intoxication reactions. Based on these findings, it is recommended to consider using intravenous lidocaine as an optional adjuvant agent to propofol-based sedation for elderly patients undergoing painless gastroscopy.

## Data Availability

The datasets generated during and analyzed during the current study are available from the corresponding author on reasonable request.

## Abbreviations

BMI: Body mass index
ASA: American Society of Anesthesiologists
MOAA/S: Modified Observer’s Assessment of Alertness/Sedation Scale
PACU: Postanesthesia care unit
ED50: Median effective dose
CI: Confidence interval
MAP: Mean arterial pressure
HR: Heart rate
SpO_2_: Pulse oxygen saturation
RR: Respiratory rate
